# Exophtalmie unilatérale révélant un grand anévrysme de la carotide interne: à propos d´un cas

**DOI:** 10.11604/pamj.2021.39.196.30577

**Published:** 2021-07-13

**Authors:** Khaoula Boukili, Loubna Elmaaloum, Bouchra Allali, Asmaa Elkettani

**Affiliations:** 1 Service d´ophtalmologie, Hôpital 20 Août, Université Hassan II, Faculté de Médecine et de Pharmacie, Casablanca, Maroc

**Keywords:** Anévrysme, carotide interne, sinus caverneux, exophtalmie, à propos d’un cas, Aneurysm, internal carotid, cavernous sinus, exophthalmos, case report

## Abstract

L´anévrysme de l´artère carotide interne intra caverneuse est une pathologie rare, pourvoyeuse de complications majeures. Le tableau clinique typique est une ophtalmoplégie avec des céphalées intenses. Le diagnostic est suspecté cliniquement, l´angiographie cérébrale confirme le diagnostic et guide la prise en charge thérapeutique. Le traitement de référence est l´embolisation artérielle par voie endo vasculaire. Nous rapportons le cas d´une femme de 80 ans qui consulte aux urgences pour une exophtalmie droite et des céphalées brutales évoluant depuis 2 semaines avant sa consultation. Le diagnostic d´un anévrysme de l´artère carotide interne fissuré dans le sinus caverneux est retenu chez elle.

## Abstract (en)

Intracavernous carotid artery aneurysm is rare but it leads to major complications. Common clinical picture is characterized by ophthalmoplegia associated with intense headaches. Cerebral angiography is used to confirm clinical suspicion and it guides therapeutic management. Endovascular embolization is the standard treatment. We here report the case of a 80-year-old woman presenting to the emergency room with right-sided exophthalmos and severe headaches occurring 2 weeks before her visit. The diagnosis of fissurized intracavernous carotid artery aneurysm was retained.

## Introduction

L´anévrysme de l´artère carotide interne au niveau de sa portion intra caverneuse est une pathologie rare, pourvoyeuse de sévères complications fonctionnelles voire même vitales. Le sinus caverneux est le lieu de passage d´un important paquet vasculaire et nerveux à destination orbitaire, ainsi, ces anévrysmes se manifestent par une symptomatologie ophtalmologique riche dominée par les paralysies oculomotrices. L´accès chirurgical au sinus caverneux est difficile et pourvoyeur d´importantes lésions associées. La voie endo vasculaire est la voie de traitement de référence dans ces pathologies.

## Patient et observation

**Information de la patiente**: nous rapportons le cas d´une femme âgée de 80 ans, sans antécédent pathologique particulier, qui se présente aux urgences pour prise en charge d´une exophtalmie avec ptosis de l´œil droit installés une semaine avant sa consultation dans un contexte de céphalées intenses.

**Résultat clinique**: l´examen de l´œil droit trouve une acuité visuelle à perception lumineuse négative, une exophtalmie douloureuse axile non pulsatile, avec une paralysie oculomotrice dans les 9 positions du regard et un chémosis hémorragique. L´examen de la surface a objectivé la présence d´une kératite d´exposition avec d´un ulcère de cornée millimétrique en inférieur. La chambre antérieure est de profondeur normale, le réflexe photomoteur direct et consensuel sont abolis. Le tonus oculaire est à 40 mmhg. L´examen du fond d´œil objective une atrophie optique. L´examen de l´œil controlatéral est sans particularités. L´examen de la 5ème paire crânienne trouve une anesthésie du territoire de la branche ophtalmique du nerf trijumeau, le reste de l´examen neurologique est sans particularités. La mesure de la tension artérielle est normale.

**Figure 1:**
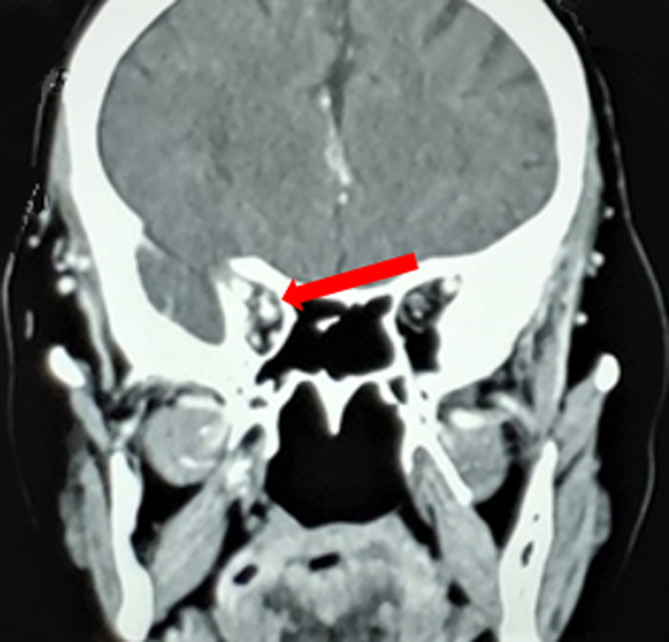
coupe scannographique frontale: anévrysme de la carotide intra caverneuse

**Figure 2:**
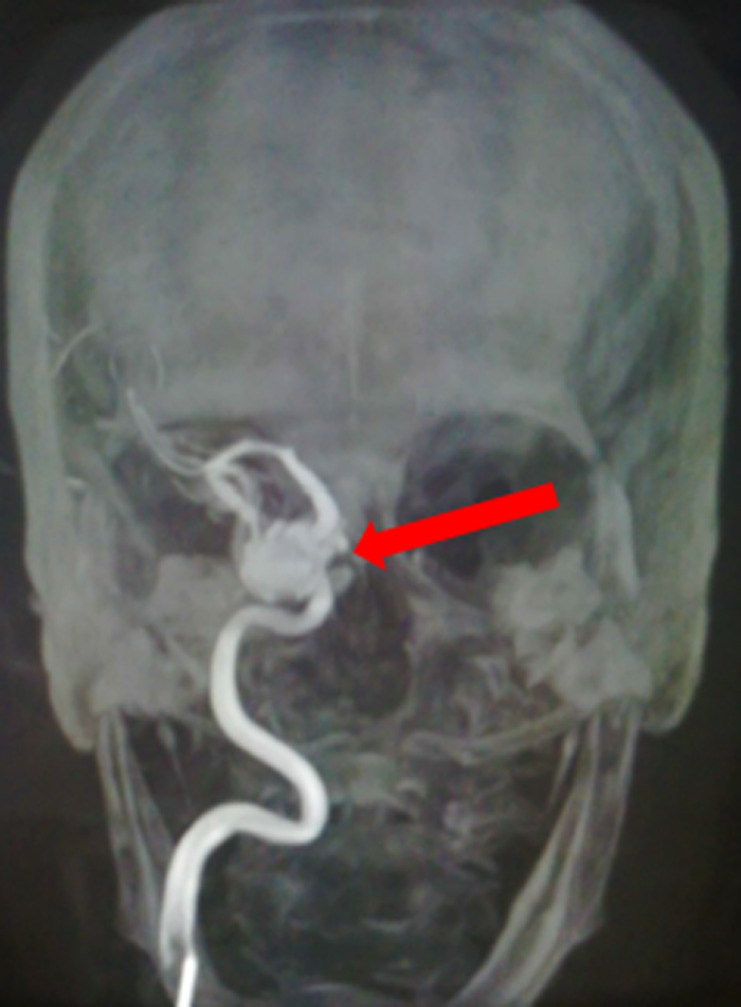
angiographie cérébrale en incidence de face: volumineux anévrysme implanté sur le bord inférieur de l'artère carotide intra caverneuse

**Démarche diagnostique**: la patiente a bénéficié d´un scanner cérébral qui a révélé l´aspect d´un anévrysme de l´artère carotide intra caverneuse droite (Figure 1). Une angiographie cérébrale est réalisée et a montré la présence d´un volumineux anévrysme mesurant 18 x 10 mm, sacciforme, implanté sur le bord inférieur de l´artère carotide intra caverneuse et qui est fissuré dans le sinus caverneux et responsable d´une compression de ce dernier avec dilatation des veines ophtalmiques homolatérales (Figure 2). Un bilan biologique général n´a pas objectivé de syndrome inflammatoire biologique.

**Figure 3:**
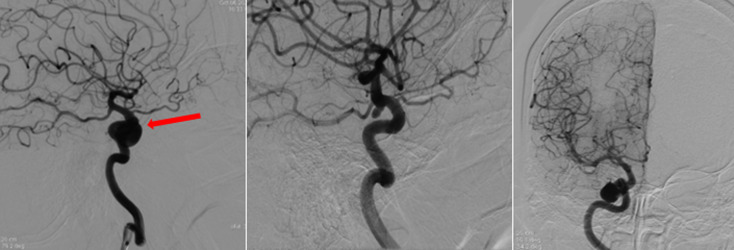
angiographie cérébrale de contrôle en incidences obliques et face: disparition du retour veineux précoce depuis le sinus caverneux vers le globe oculaire avec présence d'un anévrysme de la carotide intra-caverneuse droite

**Figure 4:**
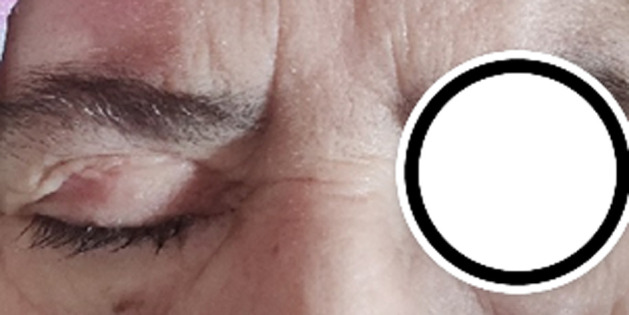
diminution de l'exophtalmie avec ptosis résiduel

**Intervention thérapeutique et suivi**: la patiente a bénéficié, dans un délai de 48 heures, d´une embolisation avec occlusion carotidienne distale droite après un test de clampage. Lors du geste, l´angiographie cérébrale a montré une diminution spontanée de la taille de l´anévrysme, une disparition de la fissure et une absence du retour veineux précoce depuis le sinus caverneux vers le globe oculaire ainsi que vers le sinus coronaire et les sinus latéraux (Figure 3). Les suites post opératoires ont été favorables avec une régression de l´exophtalmie et de la kératite d´exposition et une normalisation du tonus oculaire, par ailleurs la patiente a gardé une ophtalmoplégie complète avec un ptosis majeur (Figure 4).

## Discussion

L´anévrysme de l´artère carotide interne intra caverneuse sont extrêmement rares (3 à 5 % de tous les anévrismes intra crâniens [1]) et touchent préférentiellement les femmes d´un âge moyen de 48 ans. Ils sont souvent secondaires à une hypertension artérielle, l´étiologie traumatique et les anomalies du tissu conjonctif sont aussi évoquées [2]. Cette localisation anatomique explique la richesse de la symptomatologie ophtalmologique qui est dominée par l´ophtalmoplégie (93 %) et le ptosis (51 %) [3]. En cas de paralysie isolée, elle implique souvent le nerf abducens VI suivi du nerf oculomoteur commun III. La prise en charge thérapeutique est basée sur l´embolisation par voie endo vasculaire avec clampage de la carotide distale. La chirurgie conventionnelle est grande pourvoyeuse de complications, vu la localisation anatomique.

## Conclusion

L´anévrysme de l´artère carotide intra caverneuse est une urgence diagnostique et thérapeutique, le pronostic vital peut être mis en jeu en cas de rupture intra caverneuse.
